# Spatiotemporal risk of human brucellosis under intensification of livestock keeping based on machine learning techniques in Shaanxi, China

**DOI:** 10.1017/S0950268824001018

**Published:** 2024-10-24

**Authors:** Li Shen, Chenghao Jiang, Fangting Weng, Minghao Sun, Chenxi Zhao, Ting Fu, Cuihong An, Zhongjun Shao, Kun Liu

**Affiliations:** 1 School of Remote Sensing and Information Engineering, Wuhan University, Wuhan, China; 2 Department of Epidemiology, Ministry of Education Key Lab of Hazard Assessment and Control in Special Operational Environment, School of Public Health, Air Force Medical University, Xi’an, China; 3 Department of Plague and Brucellosis, Shaanxi Center for Disease Control and Prevention, Xi’an, China; 4 Department of Microbiology and Immunology, School of Medicine, Xi’an Jiaotong University, Xi’an, China

**Keywords:** Human brucellosis, risk prediction, machine learning techniques, impact factors

## Abstract

As one of the most neglected zoonotic diseases, brucellosis has posed a serious threat to public health worldwide. This study is purposed to apply different machine learning models to improve the prediction accuracy of human brucellosis (HB) in Shaanxi, China from 2008 to 2020, under livestock husbandry intensification from a spatiotemporal perspective. We quantitatively evaluated the performance and suitability of ConvLSTM, RF, and LSTM models in epidemic forecasting, and investigated the spatial heterogeneity of how different factors drive the occurrence and transmission of HB in distinct sub-regions by using Kernel Density Analysis and Shapley Additional Explanations. Our findings demonstrated that ConvLSTM network yielded the best predictive performance with the lowest average RMSE of 13.875 and MAE values of 18.393. RF model generated an underestimated outcome while LSTM model had an overestimated one. In addition, climatic conditions, intensification of livestock keeping and socioeconomic status were identified as the dominant factors that drive the occurrence of HB in Shaanbei Plateau, Guanzhong Plain, and Shaannan Region, respectively. This work provided a comprehensive understanding of the potential risk of HB epidemics in Northwest China driven by both anthropogenic activities and natural environment, which can support further practice in disease control and prevention.

## Introduction

Brucellosis is a widespread bacterial zoonosis caused by genus *Brucella*, which is characterized by ongoing environment-to-individual transmission globally [1, 2]. Human can be infected through direct contact with infected animals, eating contaminated animal products, or inhaling infectious aerosols [3, 4]. Currently, human brucellosis (HB) cases have been reported in over 170 countries and regions, with an annual estimate of 500 000 newly emerging cases worldwide [5]. As one of the most significantly neglected 63 zoonotic diseases, the substantial residual disability and high relapse rates of HB have caused a heavy socioeconomic burden on public health [6, 7]. In China, the number of HB cases has experienced an sharp rise over the past two decades, with northern pasturelands being the primary epidemic region, and then geographically expanding towards the grasslands, croplands and even coastal areas in Southern China [8]. Therefore, it is imperative to predict how the potential risk of HB spatiotemporally distributed, driven by a variety of factors in the context of agriculture transition. This is of great significance to effectively implement control measures and prevent epidemic transmission.

The impact of global climate change on the dynamics, distribution, and spread of infectious diseases has gained significant attention [9]. The relationship between meteorological factors and HB has been explored and air pressure, wind speed, mean temperature, and relative humidity were identified as significant impactors on the prevalence of HB. Those factors might affect the epidemic transmission by changing the activity of Brucella and the contact between livestock and humans [10]. Similarly, Yang et al. have also found air temperature, sunshine duration, precipitation, relative humidity, and evaporation play a role in the transmission of local HB with an evident lag effect. Particularly in environments with high air temperature, low relative humidity, and short sunshine duration, the risk of brucellosis infection appears higher [6]. In addition, based on a spatiotemporal analysis conducted in Inner Mongolia, averaged temperature and Normalized Differenced Vegetation Index (NDVI) were discovered to be positively correlated with the incidence of HB but a negative correlation for precipitation, relative humidity, and sunshine duration [11]. The aforementioned findings were consistent with the research by Ahmadkhani et al. who revealed that the warm months with the lowest precipitation and the highest temperature were more susceptible to the spread of HB [12].

Existing studies on epidemics prediction primarily focus on applying statistical models such as regression models [13–15], transmission dynamics models [15–17], and time series models (e.g., Exponential Smoothing Models, ARIMA and its Improved Models, Bayesian Time Series Models) [6, 18–26]. Those statistical models have advantages in improving the accuracy of epidemic risk prediction, identifying its dynamic transmission pattern, and capturing its time serial variation based on a long-term trend. However, those models demand higher-quality observational data, comply with rigorous statistical prerequisites, and simplify the complex transmissions into a similar scenario. This possibly causes poor stability of the prediction performance and limited adaptability in epidemic data of fine-grained time granularity. In addition, they are not suitable to effectively capture the epidemic dynamic process, as well as its spatiotemporal interactions with the intricate anthropogenic and natural environments [1]. Moreover, less attention was paid to integrating potential factors associated with agriculture intensification and livestock development that can increase the risk of zoonotic diseases. There is a need to reveal the geographical heterogeneity of how such anthropogenic activities affect the transmission of epidemic diseases.

In recent years, the machine learning approach has gained increasing interest within the academic community on epidemic risk assessment [27, 28]. Particularly, the combination of deep learning and spatiotemporal analysis exhibits stronger nonlinear fitting capabilities. This can lead to higher accuracy in mining vast epidemiological and environmental attribute information, providing robust support for multi-scale and real-time epidemic monitoring. Meanwhile, it can greatly reduce the uncertainty in the early warning of disease outbreaks [29].

Therefore, the aim of this study was to improve the accuracy of HB risk prediction based on machine learning approaches with three specific objectives: (1) to quantitatively examine the spatiotemporal risk of HB in Northwest China based on ConvLSTM model; (2) to compare the suitability of different machine learning models in predicting HB risk; and (3) to investigate whether the impacts of predominant driving factors on HB transmission varied within distinct geographic areas, in the context of livestock agriculture transition. This study put forward an in-depth understanding of zoonotic risk distribution in intensive livestock industries and provided valuable insights for formulating effective control strategies.

## Study area and data

### Study area

**Figure 1.**
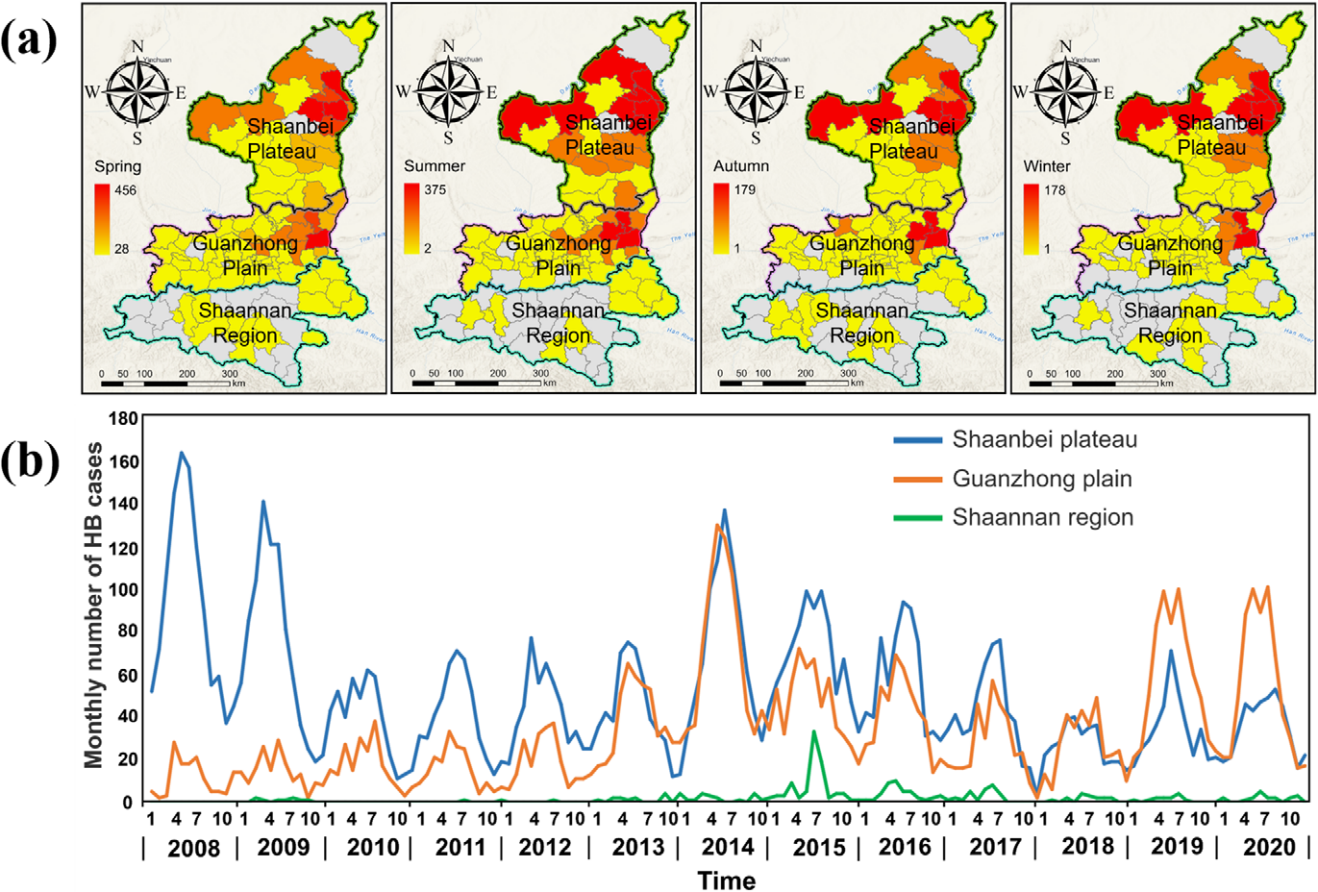
Seasonal fluctuation and geographic heterogeneity of HB incidence across three sub-regions of Shaanxi Province, 2008–2020. (a) Scenario maps based on season-wise and sub-region-wise information and (b) Monthly number of HB cases for three sub-regions from 2008 to 2020.

Shaanxi Province in Northern China is one of the most severely affected regions by highly prevalent brucellosis, due to an average annual HB incidence of 11.5/100 000. Especially Shaanbei Plateau and Guanzhong Plain show an aggregated distribution [1, 18]. It is located in northwest China (105°29′–115°15′E to 31°42′–39°35′N), ranking among the top 10 provinces in China over the past two decades. It comprises 107 counties and districts under the administration of 10 prefecture-level cities. With an area of approximately 205 600 km^2^ and a total population of 39.53 million in 2022 (http://en.shaanxi.gov.cn/as/), Shaanxi Province has a diverse climate and distinct landforms, characterized by high terrain in the north and south and low terrain in the middle. It is geographically stratified into three contiguous natural sub-regions: Shaanbei Plateau, Guanzhong Plain, and Shaannan Region, and HB across those three sub-regions exhibits seasonal fluctuation characteristics and geographic heterogeneity (Figure 1).

### Reported human brucellosis cases

**Figure 2.**
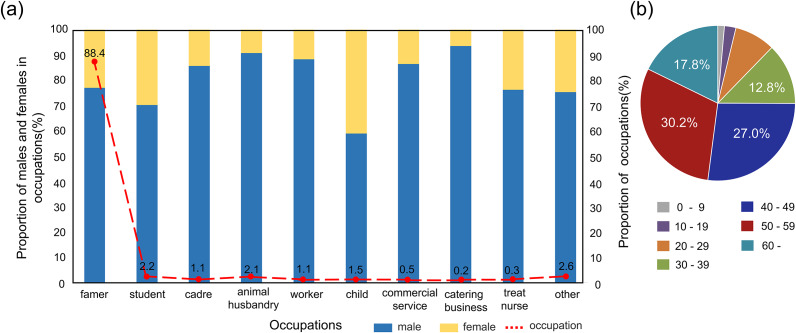
The epidemic profile of HB cases in Shaanxi Province, 2008–2020. (a) Distribution of occupation and gender and (b) Percentage of different age groups of HB (0–9, 10–19, 20–29, 30–39, 40–49, 50–59, and > 60).

The diagnosed HB cases in Shaanxi from 2008 to 2020 were provided by the Center for Disease Control and Prevention of Shaanxi Province via the National Notifiable Infectious Diseases Reporting Information System. Each HB case record contained information regarding, age, sex, occupation, and onset date of symptoms. The residential addresses were collected, and all study-related information was analyzed anonymously. The burden of HB disease in Shaanxi Province can be further demonstrated by an epidemiological profile (Figure 2). From 2008 to 2020, there were more males than females with HB in Shaanxi Province, with an incidence ratio of male to female of 3.36: 1. The dominant occupation was mainly farmers, accounting for 88.4% of the total cases (Figure 2a). The age group of 50–59 years old had the largest number of HB cases, accounting for 30.2% of the total HB occurrences (Figure 2b).

The administrative region codes were updated based on the 2020 administrative region code issued by the Ministry of Civil Affairs of the People’s Republic of China (https://www.mca.gov.cn/). These updated codes were utilized to ascertain the number of cases in each county and district. Firstly, we carefully examined the residential addresses of HFMD cases and disease onset time not during the study period, and excluded those confirmed outlier cases. Secondly, we converted the text residential address of each HFMD case into a spatial point located within the corresponding administrative boundary, and then assigned the attribute information to those points. Incomplete addresses were uniformly categorized into the geometric centre of townships. Finally, a total of 13 504 HB cases were obtained, distributed among 107 county-level administrative regions in 2020.

### Driving factors

We collected a variety of data on driving factors that potentially affect the incidence of HB, including statistical data and remote sensing products. Statistical data included population size, population density, livestock inventory (including pigs, cattle, and sheep), and meat and dairy production from 2008 to 2020, which were obtained from the Statistical Yearbook of the Shaanxi Provincial Bureau of Statistics (http://tjj.shaanxi.gov.cn/tjsj/ndsj/tjnj/). Counties and districts with no livestock inventory records were assigned a value of zero. In addition, the above annual data were sampled to improve the temporal resolution to acquire monthly data for the current year.

**Figure 3.**
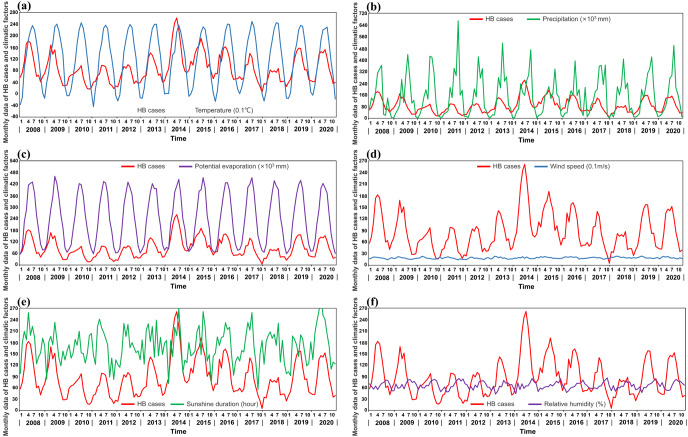
Time series XY plots of HB cases and climatic factors. (a) With temperature (0.1°C), (b) with wind speed (0.1 m/s), (c) with precipitation (×10^3^ mm), (d) with sunshine duration (hour), (e) with potential evaporation (×10^3^ mm), and (f) with relatively humidity.

We compiled monthly data on precipitation, potential evaporation, sunshine duration, average temperature (https://data.tpdc.ac.cn/home), wind speed, and relative humidity (https://cds.climate.copernicus.eu/) from 2008 to 2020 in Shaanxi Province, constituting a climate factor dataset. The time series XY plots of HB cases and climatic factors are shown in Figure 3.

In addition, to compensate for the absence of year-by-year GDP data for Shaanxi, nighttime-light remote sensing products (https://eogdata.mines.edu/products/vnl/) were used as a primary indicator of the socioeconomic level. Existing studies have proved that there is a high correlation between night light and GDP [30], and night light remote sensing products are often used to assess and monitor social and economic dynamics [31]. Relative studies also show that the expansion pattern of built-up areas is highly correlated with socio-economic factors such as GDP, per capita disposable income, population growth, industrialization and urbanization process [32]. Therefore, we used nighttime-light remote sensing products as one indicator of the socioeconomic level. The impervious surface data within the land cover dataset (https://zenodo.org/record/5816591) were obtained to estimate the built-up area, serving as another indicator of the socioeconomic level.

### Points of interest related to livestock keeping

Points of interest (POI) data of various enterprises engaged in animal husbandry were gathered from Baidu’s Aiqicha (https://aiqicha.baidu.com/) according to three rules: (1) having business records in Shaanxi from 2007 to 2020, (2) possessing detailed addresses to obtain latitude and longitude information, and (3) engaging in activities related to raising, trading, or slaughtering beef cattle, goats, sheep and cow, or selling products derived from the above animals.

## Methods

### The geospatial grid partitioning

In this study, the unit of county and district was determined as the basic spatial scale for analysis. To emphasize the local spatial relationships brought by the convolutional layer, the study area was partitioned into a grid of equally sized cells (26 × 44) while preserving the topological relationships between the counties. The observation values in the dataset were mapped to a predefined spatial region delineated by latitude and longitude coordinates.

**Figure 4.**
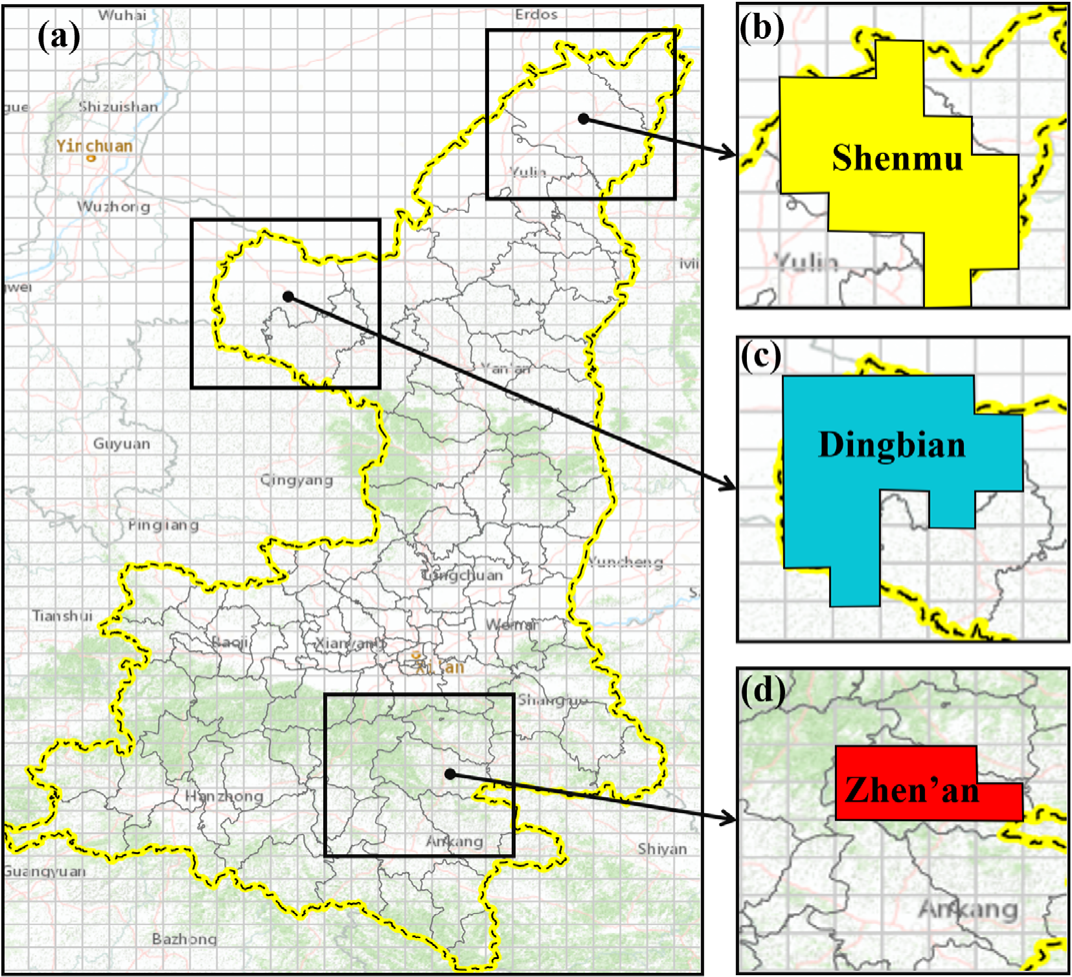
The study area is divided into the geospatial grid and merged. (a) The spatial grid with 26 × 44 cells, (b) the merged cells of Shenmu city, (c) the merged cells of Dingbian county, and (d) the merged cells of Zhen’an county.

For each cell, an administrative region code was assigned based on the following criteria: (1) Cells without any counties or districts were unallocated a code; (2) Cells containing only one county or district, which accounted for more than one-third of the cell’s area, were assigned the corresponding code; and (3) Cells encompassing multiple counties or districts were assigned the code of the county or district with the largest area. Finally, cells sharing the same administrative region code were merged, as illustrated in Figure 4. The HB case data in merged counties or districts were divided by the total number of occupied cells, yielding specific values for each cell. These geospatial grids were then transformed into raster images and normalized to the range of 0–255, in which the grey value of each pixel corresponds to a specific numerical value.

### The PCA-based ConvLSTM network

In this study, a principal component analysis (PCA) method is utilized to address multicollinearity among the feature variables. The PCA effectively reduced the dimensionality of the sample by transforming the original variables into a comprehensive set of independent variables, enabling the extraction of crucial information from multidimensional features [33].

The Convolutional Long Short-Term Memory (ConvLSTM) [34] is a variant of the Long Short-Term Memory (LSTM) model [35] that incorporates convolutional operations within the recurrent architecture. This integration enables ConvLSTM to effectively capture both spatial and temporal dependencies in sequential data, making it particularly suitable for predicting spatiotemporal patterns.

When dealing with multidimensional data, LSTM struggles to effectively capture its spatial correlations and features. To remedy this, the ConvLSTM layer swaps out the matrix multiplication of LSTM for convolution operations, improving its performance with multidimensional data. The explicit expression of a unit of ConvLSTM is given in equations (1))–(5):(1)

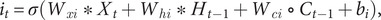


(2)

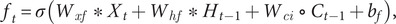


(3)

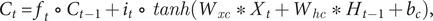


(4)

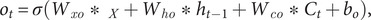


(5)

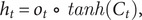

 where *i, f, c*, and *o* to are respectively referred to as the input gate, forget gate, control unit, and output gate. The weight matrices 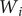 , 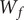 , 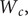 and 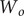 are the weights from the input to output gate. 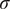 is a logistic sigmoid multiplication function with an output range of [0,1]; tan*h* denotes a hyperbolic tangent function with an output range of [−1,1]. 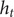 represents the output value at time 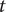 and 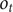 denotes the gate control information in the output gate. 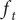 is a control function that determines which parts of the historical information should be discarded, that is, the influence of the information in the previous memory cell 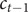 on the current memory cell 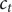 . 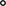 represents the Hadamard product, and 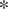 is a convolution operator.

We trained the ConvLSTM network using the variables that have undergone PCA and refer to this approach as the PCA-based ConvLSTM network. In this study, we utilized SPSS 27.0 software for conducting principal component analysis and implemented the models using Python 3.8.

Since data were initially divided into three geographical regions, the northern Shaanxi Plateau, the central Shaanxi Plain, and the southern Shaanxi region, we accordingly further derived both training and testing sets randomly based on time. Specifically, data from the first 11 years was used for model training and data from the last 2 years for model testing. However, due to the policy of data accessibility, we are unable to obtain a large dataset enough to create a separate validation set. Therefore, the cross-validation was not performed in this study as the relatively small data set did not support effective cross-validation. Otherwise, the small number of training samples may affect the stability and generalization ability of the models. In addition, the current analysis has undergone rigorous preprocessing and feature engineering, which can provide relatively high accuracy and reliability of the final results.

In our study, due to the small dataset size (only 156 samples) and limited adjustable parameters (no more than four per model), we opted for manual adjustment of certain parameters instead of automatic optimization. Specifically, for the ConvLSTM model, we manually adjusted the time step and convolutional kernel size; for the LSTM model, we manually adjusted the time step; and for the random forest model, we tuned parameters including the number of decision trees and maximum tree depth.

### Model performance evaluation metrics

The root mean square error (RMSE) and mean absolute error (MAE) are used to evaluate the fitting and predictive performance of the models, which are modelled as:(6)

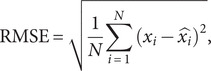


(7)

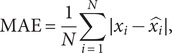

where 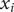 denotes the actual values, 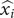 is predicted values, 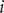 = 1, 2, …, n is the number of samples and 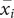 refers to the mean of actual values. RMSE and MAE serve as metrics to assess the deviation and discrepancies between predicted and actual values, with smaller values indicating higher model fitting and accuracy, respectively.

### Shapley additional explanations method

This study employed Shapley Additional Explanations (SHAP) to measure the correlation between each feature of the sample and its prediction results [36]. SHAP is a machine learning interpretive method based on a mathematical model and is a method of interpretation that is based on game theory, using the Shapley value to evaluate the contribution of each feature to the model output. The SHAP value obeys the following formula (8):(8)

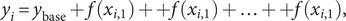

where 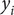 represents the predicted value for sample 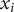 , 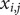 is the *j*th feature of the sample 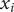 , 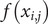 denotes the SHAP value of 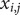 , and 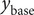 is usually the average of the observed values for all samples. When 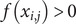 , it indicates that the feature 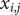 plays a positive role in the prediction of the observed value. On the contrary, the feature has the opposite effect on the prediction of the observed value.

## Results

### Predictive performance for Shaanbei Plateau

To visually present the prediction results, the counties and districts in Shaanxi were divided into three sub-regions including Shaanbei Plateau, Guanzhong Plain, and Shaannan Region. The prediction performance of RF and LSTM models was compared to that of ConvLSTM network (Table 1).

**Table 1.** The prediction performance metrics of both training and testing data for three models across different sub-regions

Sub-region	Model	RMSE (train)	MAE (train)	RMSE (test)	MAE (test)
Shaanbei	RF	8.525	5.642	9.764	6.500
	LSTM	8.019	6.002	9.287	6.167
	ConvLSTM	6.948	5.163	7.635	5.875
Guanzhong	RF	15.385	12.154	17.106	14.292
	LSTM	17.235	12.072	19.164	14.500
	ConvLSTM	13.103	11.327	16.719	12.958
Shaannan	RF	1.031	1.003	1.339	1.125
	LSTM	1.004	0.738	1.275	0.875
	ConvLSTM	0.932	0.740	1.208	0.958

**Figure 5.**
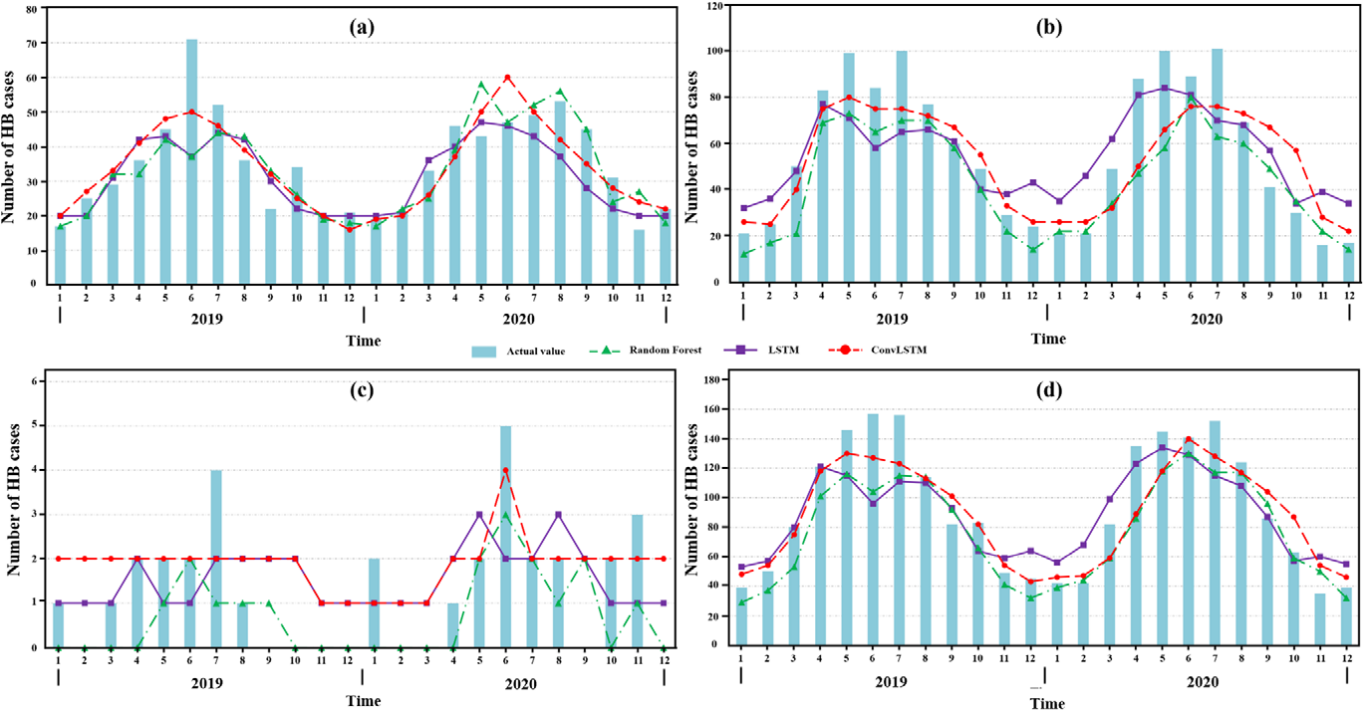
Monthly forecasted results of the PCA-based ConvLSTM, Random Forest, and LSTM models with the actual number of HB cases, 2019–2020. (a) Shaanbei Plateau, (b) Guanzhong Plain, (c) Shaannan Region, and (d) Shaanxi Province.

Shaanbei Plateau exhibited the highest concentration of HB cases in Shaanxi. Figure 5a demonstrated that all of those three models captured the temporal fluctuations in HB cases of Shaanbei Plateau from 2019 to 2020. Notably, ConvLSTM (RMSE = 9.764, MAE = 6.500) outperformed RF (RMSE = 7.635, MAE = 5.875) and LSTM (RMSE = 9.287, MAE = 6.167). Particularly during the outbreak month of June 2019, the predicted value of ConvLSTM was closer to the actual value compared to the other two models. However, the prediction performance of all those three models declined in 2020.

### Predictive performance for Guanzhong Plain and Shaannan Region

The predictive performance for Guanzhong Plain was inferior to that for Shaanbei Plateau due to higher values of RMSE and MAE. In longitudinal comparison, ConvLSTM (RMSE = 16.719, MAE = 12.958) still performed the best among those three models (Figure 5b). Moreover, the performance of LSTM (RMSE = 19.164, MAE = 14.500) was inferior to that of RF (RMSE = 17.106, MAE = 14.292). The number of HB cases in the Shaannan region appeared much lower than in Shaanbei Plateau and Guanzhong Plain, and all of these three models failed to effectively capture the temporal variations of the disease prevalence in the Shaannan region (Figure 5c).

### Predictive performance for the whole Shaanxi Province

**Figure 6.**
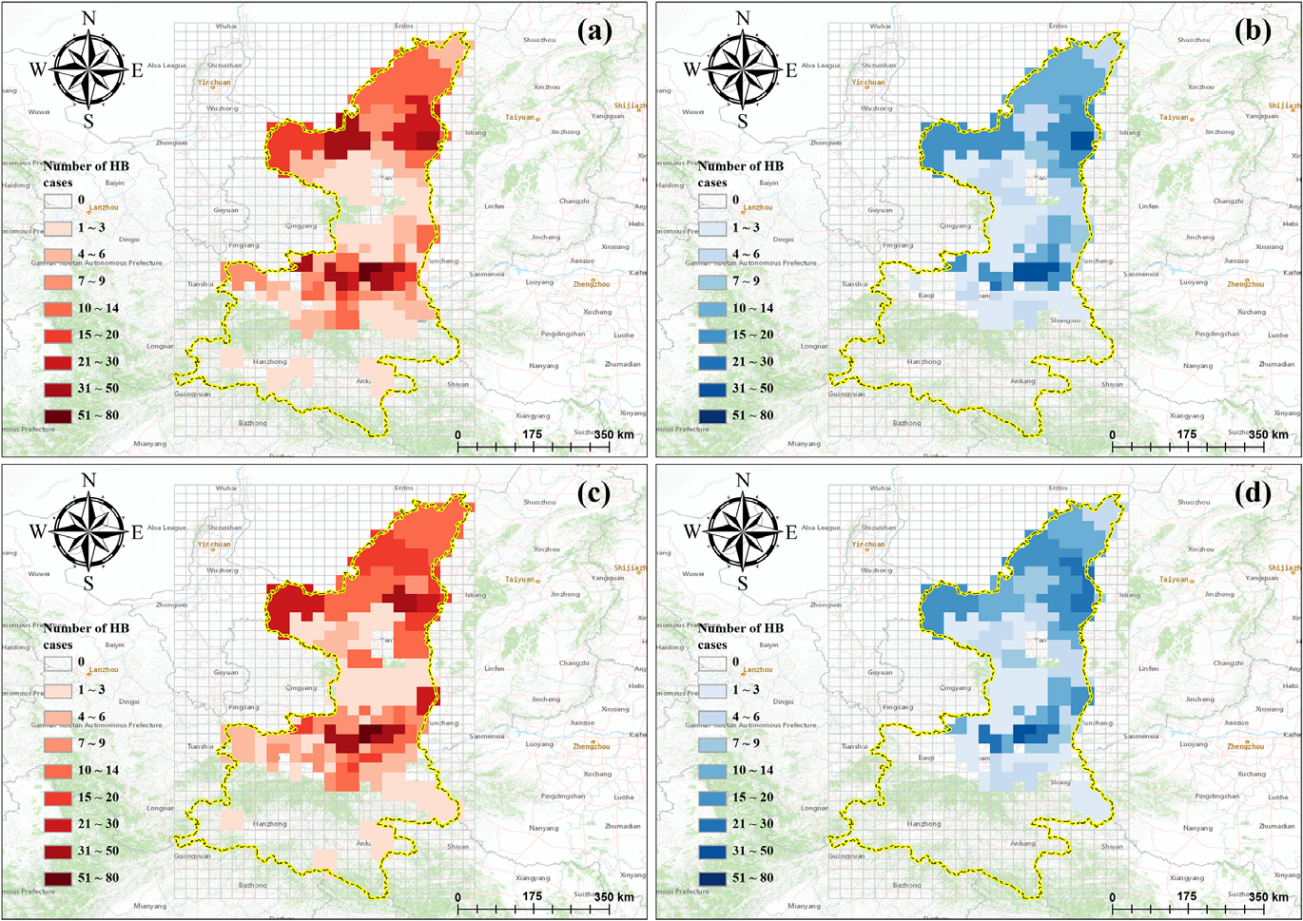
The actual and forecasted distribution of the cumulative number of HB cases in Shaanxi. (a,c) Actual distribution from April to September 2019 and 2020 and (b,d) forecasted distribution from April to September 2019 and 2020.

We aggregated the results of each sub-region to obtain the overall predictive performance for the entire Shaanxi Province (Figure 5d), and the cumulative actual and predicted number of HB cases from April to September 2019 and 2020 were respectively visualized. The analysis revealed that all those three models were able to predict the general spatiotemporal changes in the number of HB cases. In addition, the ConvLSTM outperformed the other two models in terms of prediction accuracy, as it yielded the lowest average RMSE of 13.875 (Figure 6).

### Assessment of driving factors across three sub-regions

**Figure 7.**
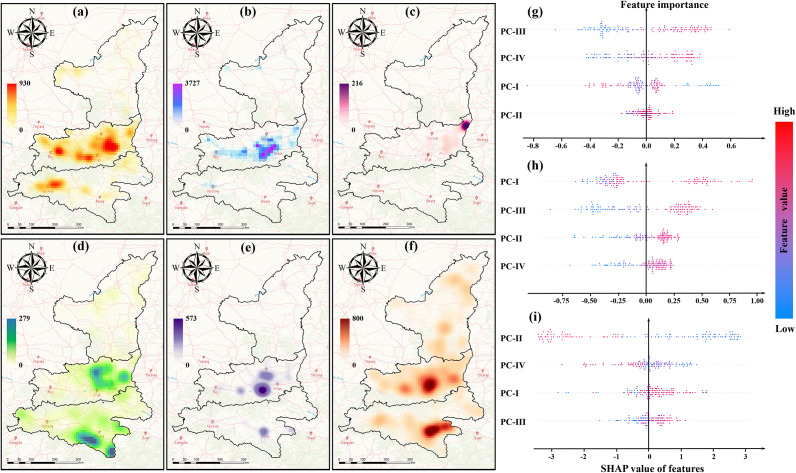
Maps of kernel density analysis of various enterprises engaged in animal husbandry in Shaanxi, 2007–2020, and the impacts of various features on the number of HB cases in sub-regions. (a) beef cattle, (b) dairy, (c) sheep, (d) goat, (e) cow, (f) comprehensive animal husbandry enterprises, (g) Shaanbei Plateau, (h) Guanzhong Plain, and (i) Shaannan Region.

We identified four principal components for each sub-region, including social economy, animal husbandry, meteorology I, and meteorology II (Table 2), which passed the *KMO (Kaiser-Meyer-Olkin)* and Bartlett’s sphericity tests. The *SHAP (Shapley Additive Explanation)* value analysis for three sub-regions is shown in Figure 7g–i.

**Table 2.** Principal components of the driving factors in Shaanbei Plateau, Guanzhong Plain, and Shaannan Region, respectively

Sub-region	PC	Category	Factors
Shaanbei Plateau	I	Social economy	BA, PpD, PpC, NL
	II	Animal husbandry	Meat, Dairy, Stock of Pig, Cattle, and Sheep
	III	Meteorology I	Tmp, Pre, PEt
	IV	Meteorology II	SD, RH, WnS
Guanzhong Plain	I	Animal husbandry	Meat, Dairy, Stock of Pig, Cattle, and Sheep
	II	Social economy	BA, PpD, PpC, NL
	III	Meteorology I	Tmp, Pre, PEt
	IV	Meteorology II	SD, RH, WnS
Shaannan Region	I	Animal husbandry	Meat, Dairy, Stock of Pig, Cattle, and Sheep
	II	Social economy	BA, PpD, PpC, NL
	III	Meteorology I	Tmp, Pre, PEt
	IV	Meteorology II	SD, RH, WnS

BA, built area; NL, nighttime-light; PC, principal component; PEt, potential evaporation; PpC, population counts; PpD, population density; Pre, precipitation; RH, relative humidity; SD, sunshine duration; Tmp, temperature; Wns, wind speed.

In Shaanbei Plateau (Figure 7g), it was found that the most important factor was PC-III, which included temperature, precipitation, and potential evaporation, closely related to climate change and exhibiting a significant positive correlation with the number of HB cases. PC-IV also played a critical role in the changes of the HB epidemic in Shaanbei Plateau, consisting of sunshine duration, relative humidity, and wind speed, which promote the spread of HB. In addition, PC-I, which represented the level of urbanization and human activities, had a certain inhibitory effect on the spread of brucellosis in Shaanbei Plateau. Surprisingly, the results of this study showed that the PC-II representing the livestock industry did not become a major influencing factor.

In Guanzhong Plain (Figure 7h), PC-I showed a positive correlation with the local HB trend, which was related to animal husbandry. Moreover, the impact of urbanization level (PC-III) on HB in the Guanzhong Plain differed significantly from that in the Shaanbei Plateau, which may have been related to the different urbanization processes and human activities in those two regions. Comparatively, meteorological features (PC-II and PC-IV) were not the main factors causing the change in HB in Guanzhong Plain. In Shaannan Region (Figure 7i), PC-II, associated with the level of socioeconomic development, significantly contributed to the distribution pattern of endemic diseases, while PC-IV, related to sunshine duration, relative humidity, and wind speed, plays a secondary role in promoting the same.

## Discussion

As an interdisciplinary research, this study attempts to predict the spatiotemporal risk of potential HB outbreaks in Northwest China, over a period of 13 years from 2008 to 2020. We also examined the driving effects of primary impact factors on the prevalence of HB across three sub-regions, from both anthropogenic and natural environmental aspects in the context of livestock husbandry intensification.

Firstly, we evaluated and compared the suitability of different machine learning models (Random Forest, LSTM, and ConvLSTM) in predicting the spatiotemporal risk of HB outbreaks. The analysis revealed that PCA-based ConvLSTM network yielded the best predictive performance owing to the lowest average RMSE of 13.875 and MAE values of 18.393. In contrast, compared to the true value RF model generated an underestimated outcome, while LSTM model had an overestimated one, particularly during the period from November 2019 to March 2020. In general, the ConvLSTM network showed superiority than Random Forest and LSTM model in predicting HB risk. This is consistent with the conclusions by previous studies conducted in Europe [37].

Moreover, we found the spatial heterogeneity of how multiple factors influence the occurrence of HB across sub-regions. For Shaanbei Plateau, as a climate-sensitive epidemic [38], the occurrence of HB was further proved to have a strong association with meteorological factors. This is consistent with the findings by Liu et al. [39], and both studies pointed out that increasing temperature can facilitate the proliferation and spread of Brucella, especially in late spring and early summer. Also, strong evaporation can increase the aridity of soil and assist the pathogen to spread into the air [40]. Also, climate factors including sunshine duration, relative humidity, and wind speed factors were all evidently associated with the potential spatiotemporal risk of HB due to the increasing transmission rate of pathogens in the open air, which aligns with the previous study conducted by Yang et al. [6] and Zheng et al. [41]. This is mainly because Shaanbei Plateau is famous for sheep and goat farming, and higher temperature can facilitate the husbandry activities of animal husbandry such as delivering, shearing, breeding, producing dairy and meat products [42]. Thus, the rising exposure to contaminated animals and their products can cause growing risk of HB transmission in Shaanbei Plateau.

Guanzhong Plain is an example of an emerging endemic area of HB, and experienced a more drastic urbanization process with intensive land use changes, which may affect the seasonal fluctuation and climate conditions. However, climate factors were identified not to be the key determinants of the transmission of HB but can still pose a significant impact in Guanzhong Plain. Higher temperature can cause a positive impact on HB occurrence, and this can possibly cause susceptible animals infected and pathogenic bacteria spread under insufficient protection and attention. This explanation matches with the explanation by Lee et al. [43]. In Guanzhong Plain, compared to climate factors, animal husbandry plays the dominant role in driving the emergence and transmission of HB through frequent livestock (cattle, sheep, and pigs) transportation and trades, which can be attributed to the growing demand for dairy and meat products by a large population in central Shaanxi. Support for this explanation also comes from previous studies by Yang et al. [6] and Chen et al. [44]. In the complex process chain of animal husbandry production, livestock, veterinary medicine, by-products, and other agents may act as the transmission route of Brucella, especially under intensive agriculture industrialization and feed pollution [45].

For Shaannan Region, characterized by comparatively geographical disadvantages and socioeconomic underdevelopment, sporadic HB cases were reported in recent years. It was found that both climate factors and livestock development were likely to affect the occurrence and transmission of HB. In Shaannan, the duration of sunshine, relative humidity, and wind speed were negatively correlated with the HB epidemic, which may have explained the lower number of HB cases in this area compared to the other two regions. It is very interesting that urbanization level in both Shaanbei Plateau and Shaannan Region is negatively associated with the occurrence of HB compared to Guanzhong Plain [46]. This can be explained by the large flow of people in economically developed areas (Guanzhong Plain) were more likely to frequently contact and infected through the consumption of contaminated food or the use of items carrying the pathogen [47]. In less developed and underpopulated areas (Shaanbei Plateau and Shaannan Region), urbanization process appeared to exert a counteractive influence on the prevalence of epidemics.

In general, the dominant factors influencing HB distribution in the three regions of Shaanxi Province were different: climate factors in Shaanbei mainly facilitates the spread of HB, while livestock development in Guanzhong primarily contributes to its transmission. Both climate factors and socioeconomic development level in Shaannan Region restricted the expansion of HB.

The contribution of this study is threefold. Firstly, distinct from the conventional statistical modelling approach, we compared different machine learning models in predicting the potential risk of HB. This improves the accuracy and allows for better utilizing the advantages of each model from a spatiotemporal perspective. Secondly, we took account of multiple influential factors into characterize the driving effects of both meteorological conditions and anthropogenic activities. This can provide a comprehensive understanding of how HB expands and transmits in the context of animal husbandry intensification. Furthermore, as a practical example of exploring how different impact factors drive the occurrence and transmission of an epidemic from a more specific scenario, this study presents some new perspectives to support formulating more effective public health strategies.

This study also has several limitations as well. Firstly, there still exists some HB cases of asymptomatic carriers unable to be surveilled and reported. Particularly, tourism and travelling are identified as important driving factors to impact the transmission of HB, but currently, it is difficult to obtain such data with enough spatial and temporal information. In further research, multiple spatial data sources (e.g., social media data, volunteer survey data, and high spectral remote sensing information) need to be enriched for providing more useful factors such as dietary habits, tourism and travelling. In addition, the neighbouring brucellosis-endemic regions of Shaanxi Province (e.g., Shanxi, Innermoglia, Hebei, and Gansu) may also contribute to the cross-region transmission and risk redistribution, which necessitates interdisciplinary and cross-regional cooperative studies. Finally, due to the data access policy, we did not obtain enough data to create a separate dataset for cross-validation and automatic parameter optimization which are critical for developing a robust predictive model.

## Conclusions

To conclude, this study examined the suitability of machine learning models in spatiotemporally predicting human brucellosis under livestock husbandry intensification, and revealed the driving effects of different factors by considering the spatial heterogeneity. To effectively prevent and control HB in Shaanxi Province, strict supervision of the livestock living environment and the management of animal husbandry practices need to be improved.

## Data Availability

The original research data for this paper can be obtained by sending a request email to the corresponding author (liukun5959@qq.com).

## References

[1] An C, et al. (2023) Exploring risk transfer of human brucellosis in the context of livestock agriculture transition: A case study in Shaanxi, China. Frontiers in Public Health 10, 2–15. 10.3389/fpubh.2022.1009854. PMC9911661 36777766

[2] Li M, et al. (2017) Model-based evaluation of strategies to control brucellosis in China. International Journal of Environmental Research and Public Health 14, 1–15. 10.3390/ijerph14030295. PMC5369131 28287496

[3] Shen L, et al. (2023) Synergistic driving effects of risk factors on human brucellosis in Datong City, China: A dynamic perspective from spatial heterogeneity. Science of the Total Environment 894, 1–9. 10.1016/j.scitotenv.2023.164948. 37336414

[4] Battikh H, et al. (2021) Clinical and laboratory features of brucellosis in a university hospital in Tunisia. Infectious Diseases Now 51, 547–551. 10.1016/j.idnow.2021.03.005. 33766736

[5] Pappas G, et al. (2006) The new global map of human brucellosis. Lancet Infectious Diseases 6, 91–99. 10.1016/S1473-3099(06)70382-6. 16439329

[6] Yang Z, et al. (2020) Spatiotemporal expansion of human brucellosis in Shaanxi Province, northwestern China and model for risk prediction. PeerJ 8, 1–17. 10.7717/peerj.10113. PMC7580622 33133781

[7] Dadar M, Shahali Y and Whatmore AM (2019) Human brucellosis caused by raw dairy products: A review on the occurrence, major risk factors and prevention. International Journal of Food Microbiology 292, 39–47. 10.1016/j.ijfoodmicro.2018.12.009. 30572264

[8] Yang H, et al. (2020) Epidemiological characteristics and spatiotemporal trend analysis of human brucellosis in China, 1950–2018. International Journal of Environmental Research and Public Health 17, 1–15. 10.3390/ijerph17072382. PMC7178157 32244493

[9] Baker RE, et al. (2022) Infectious disease in an era of global change. Nature Reviews Microbiology 20, 193–205. 10.1038/s41579-021-00639-z. 34646006 PMC8513385

[10] Cao L, et al. (2020) Relationship of meteorological factors and human brucellosis in Hebei province, China. Science of the Total Environment 703, 1–8. 10.1016/j.scitotenv.2019.135491. 31740063

[11] Peng R, et al. (2022) Driving effect of multiplex factors on human brucellosis in high incidence region, implication for brucellosis based on one health concept. One Health 15, 1–11. 10.1016/j.onehlt.2022.100449. PMC9754936 36532675

[12] Ahmadkhani M, et al. (2017) Space-time analysis of human brucellosis considering environmental factors in Iran. Asian Pacific Journal of Tropical Disease 7, 257–265. 10.12980/apjtd.7.2017D6-353.

[13] Sun W, et al. (2021) Effects and interaction of meteorological factors on hemorrhagic fever with renal syndrome incidence in Huludao City, northeastern China, 2007–2018. PLoS Neglected Tropical Diseases 15, 1–14. 10.1371/journal.pntd.0009217. PMC7993601 33764984

[14] Chen Z, et al. (2020) Prediction of hot spot areas of hemorrhagic fever with renal syndrome in Hunan Province based on an information quantity model and logistical regression model. PLoS Neglected Tropical Diseases 14, 1–16. 10.1371/journal.pntd.0008939. PMC7785239 33347438

[15] Xiao H, et al. (2013) Atmospheric moisture variability and transmission of hemorrhagic fever with renal syndrome in Changsha City, mainland China, 1991–2010. PLoS Neglected Tropical Diseases, 7, 1–7. 10.1371/journal.pntd.0002260. PMC3674989 23755316

[16] Tian H, et al. (2017) Anthropogenically driven environmental changes shift the ecological dynamics of hemorrhagic fever with renal syndrome. PLoS Pathogens 13, 1–19. 10.1371/journal.ppat.1006198. PMC5302841 28141833

[17] Li Y, et al. (2019) Intrinsic and extrinsic drivers of transmission dynamics of hemorrhagic fever with renal syndrome caused by Seoul hantavirus. PLoS Neglected Tropical Diseases 13, 1–16. 10.1371/journal.pntd.0007757 PMC6776365 31545808

[18] Peng C, et al. (2020) An estimate of the incidence and quantitative risk assessment of human brucellosis in mainland China. Transboundary and Emerging Diseases 67, 1898–1908. 10.1111/tbed.13518. 32077219

[19] Peng C, et al. (2020) Spatial-temporal distribution of human brucellosis in mainland China from 2004 to 2017 and an analysis of social and environmental factors. Environmental Health and Preventive Medicine 25, 1–14. 10.1186/s12199-019-0839-z. 31898483 PMC6941396

[20] Liang W, et al. (2018) Mapping the epidemic changes and risks of hemorrhagic fever with renal syndrome in Shaanxi Province, China, 2005–2016. Scientific Reports 8, 1–10. 10.1038/s41598-017-18819-4. 29335595 PMC5768775

[21] Yu L, et al. (2016) ARIMA model analysis of the epidemic characteristics of hemorrhagic fever with renal syndrome and meteorological factors in the Guangdong Region. Chinese Journal of Disease Control 20, 851–855. 10.16462/j.cnki.zhjbkz.2016.08.024 (in Chinese).

[22] Hu B, et al. (2020) Integration of a Kalman filter in the geographically weighted regression for modeling the transmission of hand, foot and mouth disease. BMC Public Health 20, 1–15. 10.1186/s12889-020-08607-7. 32276607 PMC7146977

[23] He J, et al. (2019) Probabilistic logic analysis of the highly heterogeneous spatiotemporal HFRS incidence distribution in Heilongjiang province (China) during 2005–2013. PLoS Neglected Tropical Diseases 13, 1–28. 10.1371/journal.pntd.0007091. PMC6380603 30703095

[24] Zhao Y, et al. (2018) A new seasonal difference space-time autoregressive integrated moving average (SD-STARIMA) model and spatiotemporal trend prediction analysis for hemorrhagic fever with renal syndrome (HFRS). PLoS One 13, 1–20. 10.1371/journal.pone.0207518. PMC6261020 30475830

[25] Sun L and Zou L (2018) Spatiotemporal analysis and forecasting model of hemorrhagic fever with renal syndrome in mainland China. Epidemiology and Infection 146, 1680–1688. 10.1017/S0950268818002030. 30078384 PMC9507955

[26] Bagheri H, et al. (2020) Forecasting the monthly incidence rate of brucellosis in west of Iran using time series and data mining from 2010 to 2019. PLoS One 15, 1–18. 10.1371/journal.pone.0232910. PMC7217463 32396582

[27] Wang Y, Shen Z and Jiang Y (2018) Comparison of ARIMA and GM(1,1) models for prediction of hepatitis B in China. PLoS One 13, 1–11. 10.1371/journal.pone.0201987. PMC6122800 30180159

[28] Alfred R and Obit JH (2021) The roles of machine learning methods in limiting the spread of deadly diseases: A systematic review. Heliyon 7, 1–12. 10.1016/j.heliyon.2021.e07371. PMC8219638 34179541

[29] Lu K, et al. (2018) Short-term wind power prediction model based on encoder-decoder LSTM. IOP Conference Series Earth and Environmental Science 186, 1–7. 10.1088/1755-1315/186/5/012020.

[30] Zhao M, et al. (2017) GDP spatialization and economic differences in South China based on NPP-VIIRS nighttime light imagery. Remote Sensing 9, 1–20. 10.3390/rs9070673.

[31] Bennett MM, et al. (2017) Advances in using multitemporal night-time lights satellite imagery to detect, estimate, and monitor socioeconomic dynamics. Remote Sensing of Environment 192, 176–197. 10.1016/j.rse.2017.01.005.

[32] Wu K, et al. (2012) Land use dynamics, built-up land expansion patterns, and driving forces analysis of the fast-growing Hangzhou metropolitan area, eastern China (1978–2008). Applied Geography 34, 137–145. 10.1016/j.apgeog.2011.11.006.

[33] Pei C (2014) *Research on Principal Component Analysis and Its Application in Feature Extraction.* Dissertation, Shaanxi Normal University, 65pp. (in Chinese).

[34] Shi X, et al. (2015) Convolutional LSTM Network: A machine learning approach for precipitation nowcasting. In Proceedings of the 28th International Conference on Neural Information Processing Systems. Cambridge: MIT Press, pp. 802–810.

[35] Hochreiter S and Schmidhuber J (1997) Long short-term memory. Neural Computation 9, 1735–1780. 10.1162/neco.1997.9.8.1735. 9377276

[36] Lundberg SM and Lee S (2017) A unified approach to interpreting model predictions. Advances in Neural Information Processing Systems 30, 4768–4777. https://dl.acm.org/doi/10.5555/3295222.3295230.

[37] Shen L, et al. (2022) Predicting the spatial-temporal distribution of human brucellosis in Europe based on convolutional long short-term memory network. Canadian Journal of Infectious Diseases and Medical Microbiology 2022, 1–11. 10.1155/2022/7658880. PMC9365592 35967090

[38] Xiang J, et al. (2018) Impact of meteorological factors on hemorrhagic fever with renal syndrome in 19 cities in China, 2005-2014. Science of the Total Environment 636, 1249–1256. 10.1016/j.scitotenv.2018.04.407. 29913587

[39] Liu K, et al. (2020) Effect of climatic factors on the seasonal fluctuation of human brucellosis in Yulin, northern China. BMC Public Health 20, 1–11. 10.1186/s12889-020-08599-4. 32299414 PMC7164191

[40] Lou P, et al. (2016) Modelling seasonal brucellosis epidemics in bayingolin mongol autonomous prefecture of Xinjiang, China, 2010–2014. BioMed Research International 2016, 1–17. 10.1155/2016/5103718. PMC5107254 27872852

[41] Zheng H, et al. (2023) Influence and prediction of meteorological factors on brucellosis in a northwest region of China. Environmental Science and Pollution Research 30, 9962–9973. 10.1007/s11356-022-22831-1. 36064850

[42] Zhu H, et al. (2017) Analysis onepidemiology and spatial-temporal clustering of human brucellosis in Fujian province, 2011–2016. Chinese Journal of Epidemiology 38, 1212–1217. 10.3760/cma.j.issn.0254-6450.2017.09.014. 28910934

[43] Lee HS, et al. (2013) Time series analysis of human and bovine brucellosis in South Korea from 2005 to 2010. Preventive Veterinary Medicine 110, 190–197. 10.1016/j.prevetmed.2012.12.003. 23276400

[44] Chen Q, et al. (2016) Epidemic characteristics, high-risk townships and space-time clusters of human brucellosis in Shanxi Province of China, 2005-2014. BMC Infectious Diseases 16, 1–10. 10.1186/s12879-016-2086-x. 27993134 PMC5165709

[45] Shen M and Shen J (2018) Evaluating the cooperative and family farm programs in China: A rural governance perspective. Land Use Policy 79, 240–250. 10.1016/j.landusepol.2018.08.006.

[46] Shen L, et al. (2022) Spatiotemporal association of rapid urbanization and water-body distribution on hemorrhagic fever with renal syndrome: A case study in the city of Xi’an, China. PLoS Neglected Tropical Diseases 16, 1–21. 10.1371/journal.pntd.0010094. PMC8782472 35007298

[47] Liang D, et al. (2021) Spatiotemporal distribution of human brucellosis in Inner Mongolia, China, in 2010–2015, and influencing factors. Scientific Reports 11, 1–8. 10.1038/s41598-021-03723-9. 34930982 PMC8688419

